# Clinical value of combined detection of Carcinoembryonic Antigen and CA125 in the diagnosis of non-small cell lung cancer combined with Malignant Pleural Effusion

**DOI:** 10.12669/pjms.40.5.7956

**Published:** 2024

**Authors:** Wanyu Yan, Yakun Li, Zhanxian Peng

**Affiliations:** 1 Wanyu Yan, Department of Respiratory Medicine, Baoding No.1 Hospital, Baoding 071000, Hebei, China; 2 Yakun Li, Department of Respiratory Medicine, Baoding No.1 Hospital, Baoding 071000, Hebei, China; 3 Zhanxian Peng, Department of Respiratory Medicine, Baoding No.1 Hospital, Baoding 071000, Hebei, China

**Keywords:** Non-small cell lung cancer, Malignant pleural effusion, Carcino-embryonic antigen, CA125, Diagnostic value

## Abstract

**Objective:**

To investigate the clinical value of combined detection of carcinoembryonic antigen(CEA) and CA125 in the diagnosis of non-small cell lung cancer(NSCLC) combined with malignant pleural effusion.

**Methods:**

This was retrospective research. Fifty-six NSCLC patients combined with malignant pleural effusion in Baoding No.1 Hospital, China, from January 2020 to January 2022 were recruited as the malignant group, and another 56 NSCLC patients combined with pleural effusion in the same period were recruited as the benign group. Pleural effusion and serum specimens were collected from both groups and their carcinoembryonic antigen (CEA), carbohydrate antigen 125(CA125) and SP70 antigen levels were measured respectively. The differences in index levels between the two groups were compared, and the value of the index in diagnosing NSCLC combined with malignant pleural effusion was analyzed.

**Results:**

The positive rates of CEA, CA125 and SP70 antigen in pleural effusion were higher in the malignant group than in the benign group (*p>*0.05); The positive rates of CEA and CA125 in the malignant group were higher than those in the benign group (*p>*0.05), with no statistically significant difference between the two groups in the positive rates of SP70 antigen (*p>*0.05). ROC curve analysis revealed the value of serum CEA and CA12 in the diagnosis of NSCLC combined with malignant pleural effusion, while serum SP70 antigen had no diagnostic value (*p>*0.05).

**Conclusion:**

The combined detection of CEA, CA125 and SP70 antigen boasts a higher diagnostic value for NSCLC-mediated pleural effusion, with higher diagnostic value than the combined detection of serum indexes.

## INTRODUCTION

Pleural effusion, one of the common clinical pathologies, refers to the abnormal increase of fluid in the pleural cavity. It can be attributed to a variety of causes, such as chest disease, systemic disease, and physical injury.^1^,^2^ Clinically, drainage of pleural effusion is often the preferred option. However, due to the nature of the pleural effusion, i.e., benign or malignant, there is usually a significant difference in the prognosis of patients. Thus, the identification of benign or malignant pleural effusions is of great importance in the formulation of treatment plans.^3^,^4^ Benign pleural effusions are mostly the product of inflammatory irritation caused by diseases such as lung infection and tuberculosis. Malignant pleural effusions, by contrast, are more often generated by tumor metastases to the chest cavity, and are clinically caused by lung cancer.^5^,^6^ Cytology is a common diagnostic modality to identify the nature of pleural effusion with a high degree of specificity, but it is still prone to miss diagnosis due to specimen collection and laboratory conditions.^7^,^8^

Carcinoembryonic antigen (CEA), carbohydrate antigen 125(CA125) and SP70 antigen are tumor markers with good clinical diagnostic efficacy in the diagnosis of lung cancer. For improved efficacy in the clinical diagnosis of malignant pleural effusion associated with lung cancer, in this study, the levels of CEA, CA125 and SP70 antigens were measured in pleural effusion specimens and serum specimens from NSCLC patients combined with malignant pleural effusion in our hospital, and compared with those of NSCLC patients with pleural effusion. The diagnostic efficacy of each index in the diagnosis of NSCLC combined with malignant pleural effusion was analyzed, and the detection of two different specimens was compared, with a view to providing reference data for the clinical treatment of NSCLC combined with malignant pleural effusion.

## METHODS

This was retrospective research. Fifty-six NSCLC patients combined with malignant pleural effusion in Baoding No.1 Hospital from January 2020 to January 2022 were recruited as the malignant group, and another 56 NSCLC patients combined with pleural effusion in the same period were recruited as the benign group.

### Ethical Approval

The study was approved by the Institutional Ethics Committee of Baoding No.1 Hospital (No.: 2021101102; Date: October 11, 2021), and written informed consent was obtained from all participants.

In the malignant group, there were 36 males and 20 females, ranging in age from 48-67 years old, with an average age of (57.45±4.38) years old. In the benign group, there were 34 males and 22 females, ranging from 49-66 years old, with an average age of (57.43±3.92) years old. No statistically significant differences were observed in baseline data between the two groups (*p>*0.05).

### Inclusion criteria:

- Patients in the malignant group who were confirmed to have NSCLC by imaging, histopathology and cytology.
- Patients with tuberculous pleural effusion, or those with pleural effusion caused by a lung infection and pleural effusion caused by heart failure in the benign group.

### Exclusion criteria:

- Patients with other malignant tumors.
- Patients with immune therapy two months before specimen collection.
- Patients with vague causes of pleural effusion.
- Patients with poor compatibility when taking specimens.
- Patients with complicated asthma, pulmonary edema and other respiratory diseases.

Tuberculous pleural effusion: positive for mycobacterium tuberculosis or acid-fast staining in pleural effusion, improved after tuberculosis treatment; Pulmonary infection pleural effusion: manifesting as exudative pleural effusion, improved after anti-inflammatory treatment; Heart failure pleural effusion: suffering from cardiac insufficiency disease without other predisposing conditions, improved after intensive cardiac therapy.

All specimens were collected upon admission and stored at -20°C for testing. Pleural effusion specimens: approximately 10 ml of pleural effusion was extracted from patients under sterile conditions through B-ultrasound-guided puncture, and the supernatant was taken by centrifugation (500 r/min., five minutes). Serum specimens: fasting peripheral venous blood was collected from patients in the morning, and the supernatant was taken by centrifugation (3500 r/min., three minutes).

### Observation indexes

The CEA, CA125 and SP70 antigen levels of pleural effusion and serum specimens were measured, respectively. CEA and CA125 were measured by enzyme-linked immunosorbent assay (ELISA)(kit: Beijing Bovols Biological Technology Co., Ltd.), CEA<5.0 ng/ml was considered negative and vice versa, CA125≤35 U/ml. SP70 antigen was measured by double antibody sandwich ELISA (kit: Shenzhen HSA Biotech Co., Ltd.), with a P/N value <2.50 as negative and vice versa.

### Statistical Analysis

In this study, a database was built and SPSS 22.0 statistical software was used for statistical analysis of the data, and the measurement data satisfying normal distribution and homogeneity of variance were expressed by(*χ̅*±*S*). Two independent sample *t* test was used for comparison between groups, the count data were expressed as rates and χ^2^ test was used for the comparison of rates.

ROC curve was used to analyze diagnostic efficacy and to calculate sensitivity, specificity and kappa values. The parallel diagnosis was adopted in the combined detection, i.e., one positive of several indexes was diagnosed as positive. The Delong Test function was employed to compare the AUC between the ROC curves for the combined detection of two specimens in the diagnosis of NSCLC combined with malignant pleural effusion, with *p<*0.05 indicating a statistically significant difference.

## RESULTS

**Fig.1:**
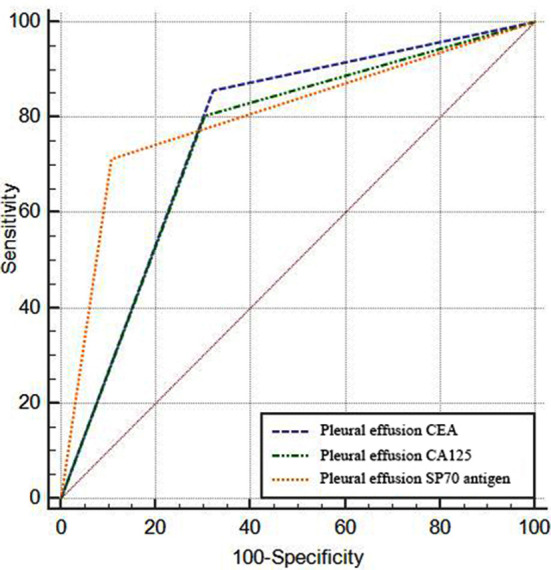
Efficacy of pleural effusion specimens in the diagnosis of NSCLC combined with malignant pleural effusion.

The positive rates of CEA, CA125 and SP70 antigen in pleural effusion were higher in the malignant group than in the benign group (*p>*0.05). Table-I. ROC curve analysis showed that CEA, CA125 and SP70 antigen in pleural effusion were all valuable in diagnosing NSCLC combined with malignant pleural effusion (*p<*0.05). At this time, CEA had an AUC of 0.768, sensitivity of 85.71%, specificity of 67.86%, accuracy of 76.79% and kappa value of 0.536; CA125 had an AUC of 0.750, sensitivity of 80.36%, specificity of 69.64%, accuracy of 75.00% and kappa value of 0.500; SP70 antigen had an AUC of 0.804, sensitivity of 71.43%, specificity of 89.29%, accuracy of 80.36% and kappa value of 0.607, Table-II and Fig.1.

**Table-I:** Comparison of the positive rate of pleural effusion specimens between the two groups (n, %).

Group	n	CEA	CA125	SP70 antigen
Malignant group	56	48 (85.71)	45 (80.36)	40 (71.43)
Benign group	56	18 (32.14)	17 (30.36)	6 (10.71)
*χ* ^2^		33.202	28.325	42.646
*P*		<0.001	<0.001	<0.001

**Table-II:** Efficacy of pleural effusion specimens in the diagnosis of NSCLC combined with malignant pleural effusion.

Tumor marker	AUC	SE	95% CI	P	Sensitivity (%)	Specificity (%)	Accuracy (%)	Kappa value
Pleural effusion CEA	0.768	0.039	0.679~0.842	<0.001	85.71	67.86	76.79	0.536
Pleural effusion CA125	0.750	0.041	0.659~0.827	<0.001	80.36	69.64	75.00	0.500
Pleural effusion SP70 antigen	0.804	0.037	0.718~0.873	<0.001	71.43	89.29	80.36	0.607

The positive rates of CEA and CA125 in the malignant group were higher than those in the benign group (*p<*0.05), with no statistically significant difference between the two groups in the positive rates of SP70 antigen (*p<*0.05), Table-III.

**Table-III:** Comparison of the positive rate of serum specimens between the two groups (n, %).

Group	n	CEA	CA125	SP70 antigen
Malignant group	56	34 (60.71)	36 (64.29)	5 (8.93)
Benign group	56	11 (19.64)	13 (23.21)	4 (7.14)
*χ* ^2^		19.651	19.193	0.121
*P*		<0.001	<0.001	0.728

**Fig.2:**
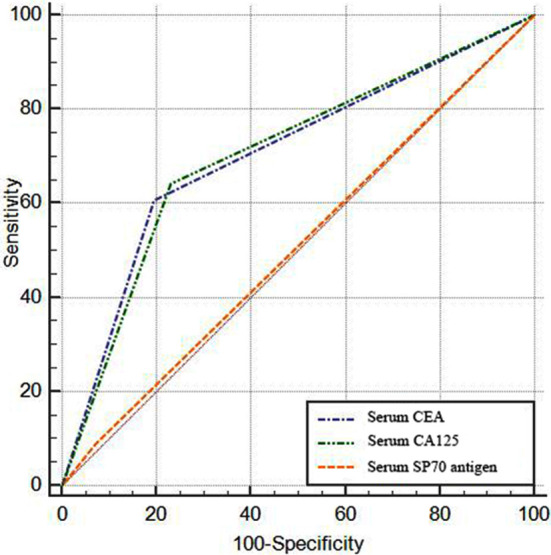
Efficacy of serum specimens in the diagnosis of NSCLC combined with malignant pleural effusion.

ROC curve analysis revealed the value of serum CEA and CA12 in the diagnosis of NSCLC combined with malignant pleural effusion (*p<*0.05). At this time, CEA had an AUC of 0.705, sensitivity of 60.71%, specificity of 80.36%, accuracy of 70.54% and kappa value of 0.411; CA125 had an AUC of 0.705, sensitivity of 64.29%, specificity of 76.79%, accuracy of 70.54% and kappa value of 0.411; While serum SP70 antigen had no diagnostic value (*p<*0.05), Table-IV and Fig.2.

**Table-IV:** Efficacy of serum specimens in the diagnosis of NSCLC combined with malignant pleural effusion.

Tumor marker	AUC	SE	95% CI	P	Sensitivity (%)	Specificity (%)	Accuracy (%)	Kappa value
Serum CEA	0.705	0.042	0.612~0.788	<0.001	60.71	80.36	70.54	0.411
Serum CA125	0.705	0.043	0.612~0.788	<0.001	64.29	76.79	70.54	0.411
Serum SP70 antigen	0.509	0.026	0.413~0.605	0.871	8.93	92.86	50.89	0.018

**Fig.3:**
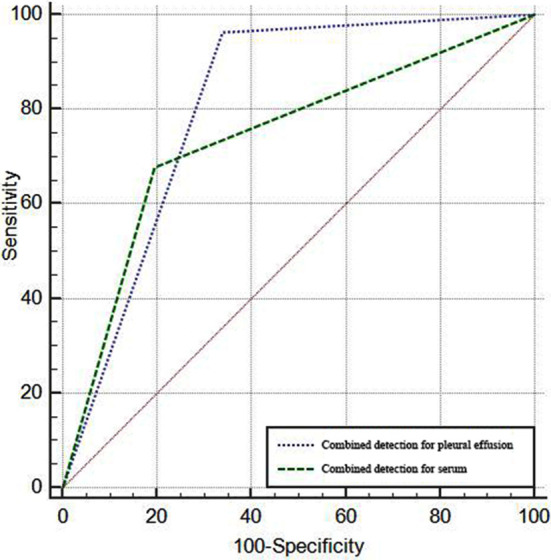
Efficacy of the combined detection of the two specimens in the diagnosis of NSCLC combined with malignant pleural effusions.

The parallel diagnosis was adopted in the combined detection. The combined detection of pleural effusion specimens detected malignant in 54 cases and benign in 37 cases, with a sensitivity of 96.43%, specificity of 66.07%, accuracy of 81.25% and kappa value of 0.625. While in the combined detection of serum specimens, 38 malignant and 45 benign cases were detected, with a sensitivity of 67.86%, specificity of 80.36%, accuracy of 74.11% and kappa value of 0.482. ROC curve analysis of both was shown in Fig.3, indicating that the efficacy of the combined detection was higher in pleural fluid specimens than in serum specimens (*p<*0.05), Table-V.

**Table-V:** Efficacy of the combined detection of the two specimens in the diagnosis of NSCLC combined with malignant pleural effusions.

Specimen	AUC	SE	95% CI
Pleural effusion	0.821	0.035	0.738~0.887
Serum	0.741	0.041	0.650~0.819
*Z*	2.299		
*P*	0.022		

## DISCUSSION

CEA, as a broad-spectrum tumor marker, is an acidic glycoprotein characteristic of human embryo antigens. It exists on the surface of cancerous cells and belongs to the structural protein of the cell membrane. It is secreted outside the cell through the cell membrane and infiltrates into the body fluids around cells. CEA has a high sensitivity in the diagnosis of malignant tumors and boasts good clinical value in the monitoring of disease conditions and the evaluation of efficacy.^9^-^11^ CA125 can be expressed in normal cells, while its expression level is low under normal conditions. When subjected to inflammatory stimulation, its level increases to assist tumor cells in immune escape and promote tumor growth and metastasis.

Studies have also shown that the increase of CA125 level can reduce the sensitivity of cancer cells to chemotherapy drugs, and then develop drug resistance, reducing the clinical therapeutic effect of cancer. Therefore, changes in CA125 levels play an important role in the malignant development of tumors.^12^,^13^ SP70 antigen exists in the cytoplasm and envelope of NSCLC cancer cells, and is a potential carcinomatous standard of NSCLC.^14^

It was shown that the antigen positive rates of CEA, CA125 and SP70 in pleural effusion specimens in the malignant group were higher than those in the benign group, indicating that CEA, CA125 and SP70 antigens were highly expressed in the malignant pleural effusion. Subsequent ROC curve analysis showed that all indexes of pleural effusion could diagnose NSCLC combined with malignant pleural effusion, which was partially consistent with previous studies.^15^-^17^

In the detection of serum samples, the positive rates of CEA and CA125 in the malignant group were higher than those in the benign group, which again confirmed the high expression of CEA and CA125 in NSCLC patients combined with malignant pleural effusion. However, there was no significant difference in the positive rate of serum SP70 antigen between the benign and malignant groups. Combined with the results of diagnostic efficacy analysis of pleural effusion samples, it suggested that SP70 antigen may be specifically expressed in NSCLC combined with malignant pleural effusion. The AUC of the combined detection of pleural effusion samples was 0.821, which was higher than that of each index separately, suggesting that the combined detection of CEA, CA125 and SP70 antigens in pleural effusion could be used to improve clinical diagnostic efficacy.

Pleural effusion, on the one hand, is caused by inflammatory irritation, such as bacterial infection, tuberculosis, and heart failure, and on the other hand, by tumor metastasis, which is often clinically caused by lung cancer. NSCLC is the most common form of lung cancer in China, and about 1/3 of NSCLC patients are initially characterized by pleural effusion.^18^,^19^ In fact, pleural effusion also has benign and malignant forms. The differentiation of benign and malignant pleural effusion is of great significance to the formulation of clinical treatment. The malignant degree of malignant pleural effusion has also a close bearing on the malignant development of the disease.^20^,^21^ Therefore, early diagnosis of NSCLC complicated with malignant pleural effusion should be made, which has important guiding significance for the early and effective intervention of patients with the disease. Previously, the diagnosis of pleural effusion was often made by pleural effusion exfoliative cytology, which has good specificity but is limited in clinical use due to the complexity of the procedure.^22^

Previous studies^23^,^24^ have revealed that tumor development stimulates the increase of the level of tumor markers, which are released into the blood, and most of them will remain in the tissue fluid around the tumor. Therefore, the positive rate of tumor markers in histological detection is higher than that in blood sample detection. This is also one of the reasons why pathological tissue tests can be used as the gold standard for the diagnosis of benign and malignant tumors. Considering the proximity of pleural effusion to the lungs, the pleural effusion and serum samples of the tested patients were used in this study to detect the levels of related tumor markers, and the effectiveness of different specimens in the diagnosis of NSCLC combined with malignant pleural effusion was analyzed.

The AUC of the combined detection of serum samples was also higher than that of the single index detection, which further confirmed the effectiveness of the combined detection of tumor markers. The comparison of the combined detection efficiency of two different specimens showed that the diagnostic efficiency of pleural effusion was significantly higher than that of serum specimen, suggesting that the extraction of pleural effusion specimen should be given priority when the exhaled cells of pleural effusion cannot be obtained. It has also been mentioned in previous studies^25^ that the combined detection of tumor markers of pleural effusion has good clinical value in the identification of NSCLC related malignant pleural effusion.

### Limitations of the study

There are still some shortcomings in this study. The number of subjects included in this study was limited, so the conclusions drawn may not be very convincing. In addition, we only analyzed and discussed the cases included in our hospital, which may not be representative enough. We look forward to a multi-center study in the future to reach more comprehensive conclusions.

## CONCLUSION

Pleural effusion and serum CEA and CA125 boast good value in the diagnosis of NSCLC combined with malignant pleural effusion, with higher value under combined detection. SP70 antigen may be specifically expressed in NSCLC combined with malignant pleural effusion, and can be combined with SP70 antigen in the detection of pleural effusion to improve clinical efficacy.

### Authors’ Contributions:

**WY:** Carried out the studies, participated in collecting data, drafted the manuscript, are responsible and accountable for the accuracy and r integrity of the work.

**YL:** Performed the statistical analysis and participated in its design.

**ZP:** Participated in acquisition, analysis, or interpretation of data and draft the manuscript.

All authors read and approved the final manuscript.
